# Making the invisible visible Normativities *in* and *of* technology assessment

**DOI:** 10.14512/tatup.28.1.21

**Published:** 2019-04-03

**Authors:** Federica Lucivero, Pierre Delvenne, Michiel van Oudheusden

**Affiliations:** Ethox Centre and Wellcome Centre for Ethics and Humanities, Nuffield Department of Population Health, University of Oxford, Old Road campus, Oxford; University of Liège, SPIRAL Research Centre; Belgian Nuclear Research Centre

**Keywords:** deliberation, ethics, normativity, pragmatism, technology assessment

## Abstract

Technology assessment (TA) is an analytic and interactive practice that produces evaluative judgments about the societal implications of technology. Despite this distinct evaluative disposition, “normativities” inherent in TA programs and practices often remain hidden. Therefore, TA practice and outcomes often overlook a range of methodological, ethical, and political issues. In an attempt to remedy this shortcoming, this article explores how TA aims to improve political decision making in science and technology (meta-normativity) and is imbued with the values, norms, and moral positions of both participants and TA practitioners (in-normativity). It provides recommendations to render these normativities in TA more visible, and thereby amenable to reconsideration and change.

## Introduction

Since the 1960s, technology assessment (TA) approaches have emerged that seek to facilitate interactions between technological experts, citizens, civil society organizations, and other relevant social groups, to enable the collective management of technology. Utilizing a range of participatory methods and techniques, TA engages these parties to deliberate towards socially robust decision outcomes and incite social learning among them. As TA seeks to be of “service to policy making and decision making more generally” (Rip 2015, p. 125), TA is normative in character. The rationale behind TA programs and processes is not just to describe the potential social implications of technologies, but also to evaluate whether these implications are good or bad (or mixed) for society. We can consider an evaluation as ‘normative’ if a technology is assessed with respect to an explicit legal or moral norm or an authoritative standard. In general terms, if the goal of TA is to facilitate the development of “better technologies in better societies” (Schot and Rip 1997, p. 256), then we may ask what is better, for whom, and why, in order to understand what TA promises and how TA contributes to science and society at large.

TA is more than an analytic activity aimed at providing decision makers with an objective analysis of a technology (van Eijndhoven 1997), and more than interactive and communicative tool that aims to enrich the basis for science and technology decision making (Decker and Ladikas 2004). Both conceptually and through its modes of operation, TA hints at a more inclusive and equitable science-society relationship than is presently the case. In addition, several contemporary TA approaches rest on a vision of democracy as a deliberative, cooperative and consensual way of dealing with social conflicts, and the conviction that social learning is morally superior to political bargaining (Abels 2007). TA therefore holds a normative and political orientation, as it invokes standards and moral principles to legitimize its procedures and guide them. Furthermore, the products of TA activities (be they recommendations, policy reports or guidelines) appeal to moral principles that the involved actors should follow, distribute roles and responsibilities among them, and favour particular solutions over others. We are hence led to question the meaning and implications of what is taken as good or bad technology development and assessment.

This normative character of TA is not often acknowledged by practitioners. In an article supporting the collaboration between the fields of ethics and TA, Grunwald (1999) argues that TA may be seen to suffer from ‘normative deficits’ (ibid., p. 174) as it does not directly engage with its evaluative goal and practice. Although several authors point out that TA has to tackle normative questions only few scholars have engaged with these questions (Grunwald 2004; Palm and Hanson 2006; Brey 2012; Lucivero et al. 2011; Lucivero 2016; Kiran et al. 2015).

*The ‘meta-normativity’ of TA is the embedded normative ideal that a more pluralistic process will produce better outcomes and benefits for society*.

In what follows, we further articulate this diagnosis by distinguishing two ways in which TA is normative. First, building on examples from TA and related literature, we discuss TA’s ‘meta-normativity’, that is, its aim to improve the process of political decision making around science and technology. We do so by articulating and critically discussing how present-day TA programs and processes engage in an ideal of democratizing decision-making processes and outcomes. Next, we explore TA’s ‘in-normativity’: TA activities are imbued with the values, norms and moralities of both participants and TA practitioners. In these activities, power relations among involved participants often come at the exclusion of discussions about what constitutes the good life. We draw lessons for TA practice that attend to the methodological, practical, and political implications for TA and the broader context in which TA plays out. In order to constructively address the criticism of the normative deficit, we provide three recommendations to render inherent normativities visible in TA processes, and thereby amenable to reconsideration and change.

### The normativity *of* technology assessment

The first type of normative deficit is widely discussed in the TA literature and concerns the democratic and participatory values that orient TA expertise (Delvenne and Parotte 2019; van Est 2019). Whereas at its origin in the 1970s, the aim of TA was to reduce the costs of technologies’ detrimental effects by anticipating potential impacts of technology and providing policymakers with neutral scientific advice, TA subsequently became a process of ongoing dialogue that supported actors’ decision-making processes. For instance, TA programs in Denmark and the Netherlands included participants and their perspectives into the process of assessing technologies (Smits et al. 1995).

When TA emerged, it was a practicable response to real-world challenges that are hard to control, such as sociotechnical uncertainties, controversies, and public ambivalence about technology development. TA pioneers sought to broaden the spectrum of issues and actors in technological decision-making within a more general cultural move towards democratization, as various counter-movements (e. g. feminists, environmentalists, pacifist, antinuclear activists, patients’ groups) in the United States and in Western Europe demanded that citizens have a say in all decisions that affect them personally (van Est and Brom 2012). It is thus against a background of political and cultural contestation and broad recognition of the inadequacy of traditional institutions to deal with the challenges posed by reflexive modernization (Hennen 1999; Delvenne et al. 2011) that TA developed as a criticism of ‘modernist’ governing generally, and of customary ways of managing technology in society specifically (Schot 2003). These two mutually reinforcing factors help explain the turn from technology *government* to technology *governance*, and from ‘expertocratic’ to interactive, participatory TA (Petermann 2000; Cruz-Castro and Sanz-Menendez 2005). By including different stakeholder groups in discussions, interactive, constructive and participatory TA approaches acknowledge the diverse and sometimes conflicting character of stakeholders’ normative positions.

The imperatives for inclusive and participatory decision-making touch upon two interrelated normative rationales, one substantive and the other procedural-democratic. While the first presents interactive decision-making as a means of achieving qualitatively better science and technology outcomes and/ or policies, the second takes interaction and inclusion as ends in themselves (Fiorino 1990). Accordingly, the first imperative is distinctly outcome- and policy-oriented, whereas the second centres on enhancing democracy and citizen/stakeholder empowerment, typically through deliberative and participatory processes. These rationales interweave in TA practice, as they reach substantively better policy outcomes through interactive and inclusive democratic processes. Furthermore, TA initiatives aim at both substantive and democratic benefits, from democratizing technology to initiating social learning and opening up opportunities for conflict resolution, among many others (Abels 2007).

As Grunwald points out (2006), at the core of TA approaches is the need for robust results by ensuring independence (the results have to be elaborated during the process and unbiased by external interests), lack of prejudice (freedom from bias and a sufficiently broad research approach), and impartiality (no preference given to certain value standpoints). However, any assessment requires some form of evaluation and is therefore normative by definition. TA’s goal of improving society or democratising decision-making around emerging technologies still requires TA outcomes to take some normative position towards some decisions. TA exercises are therefore expected to bring to the fore a diverse and plural set of values without preferring one of them. However, this neutral stance is at odds with the goal of offering an evaluation of emerging technologies, which requires by definition to support some position and take a normative stance.

The ‘meta-normativity’ of TA is the embedded normative ideal that a more pluralistic process will produce better outcomes and benefits for society (Delvenne and Parotte 2019). Here, a gap emerges between the explicitly stated normative aims of TA of democratizing science and technology decision making processes and enabling better policy outcomes on the one hand, and the actual practice of TA on the other. In fact, by involving publics in policy making processes, selective choices in the design of activities and social control of participants are required. Unless the political and moral implications of these choices are made explicit and opened to debate, there is little to no opportunity to scrutinize how they influence the deliberative process.

### The normativity *in* technology assessment

Intrinsic normativities in the TA processes and projects are also often overlooked. This happens in two ways: first, TA design is vulnerable to strategic game playing and power struggles when the divide between experts and lay people is reproduced. Second, the involvement of multiple stakeholders in an inclusive, cooperative manner does not guarantee that substantive issues about the desirability of a certain technology are taken up in the assessment. Let us look at each of these facets of ‘in-normativity’ a bit closer.

*It should be priority for TA to engage in moral issues concerning the greater good in the debate or to discuss soft impacts (intrinsic normativites)*.

In addition to the expert guided activities that characterized the dawn of TA, many parliamentary TA offices in Europe built at least part of their activities on interactive and participative methodologies. Awareness initiatives, consensus conferences, scenario workshops, citizen hearings, or deliberative mappings, among other methods, were designed and implemented by TA institutes aiming at greater experts’, stakeholders’ or citizens’ involvement (Klüver et al. 2000; Klüver et al. 2016). Although most participatory activities aim at blurring boundaries between scientific facts and social values, they have tended in practice to reinforce these distinctions (Pellizzoni 2003). For instance, by reproducing a distinct separation between experts and lay people, these mechanisms reproduce a boundary within TA: whereas citizens are called upon to discuss scientific facts in virtue of their competences in ethics and values, they are not asked or invited to criticize the way scientific facts are constructed, selected and presented (Bruun Jensen 2005; Blok 2007).

When participatory exercises are implemented, the risk is to ignore the framing, selection and contestation of the expertise and neglecting the broader political and social contexts and the reproduction of traditional power-relations between experts and lay people. As noticed in some cases of constructive TA, issues such as start-up firms and regulation dominate the discussion in stakeholder workshops (Robinson 2010), whereas in exercises with lay people issues concerning desirability of science and technology and their implications for the ‘good life’ were tackled but rarely translated into action plans (Powell and Colin 2008). Hence, it does not suffice to engage a diverse set of stakeholders in discussions to guarantee democratic interaction, choices in process design will also have some consequences on what will be addressed as important. Some interests of uninvited lay citizens from ‘the public’ may often be unlikely to have a voice when only organized groups are engaged in technology assessment.

Moreover, by shifting the focus from impacts to the process of technological innovation, stakeholder focused TA exercises may exclude some lay questions on the good life from the debate. As Richard Sclove (2010), in a report evaluating the work of the US Office for TA, points out, some values are systematically neglected in transactions and negotiations among stakeholders with specific interests. Moral issues tend to be excluded from the discussion in expert-based assessment, when there is no consideration of the possible effect of technologies on social relations in daily life, and the question of how the technologically altered quality of community relations bears, in turn, on the basic ideals, structure and functioning of a democratic society.

This point is also made by Swierstra and Molder (2012), who show how some concerns about emerging technologies raised by citizens (e. g., the question of ‘naturalness’ in food industry) are discarded or minimized by technology developers. These concerns, typically non-quantifiable and ambiguous, are considered as less important, ‘soft’ impacts that do not merit attention. If the goal of TA is to support decision-making guaranteeing democratic interactions, it should be priority for TA to engage in moral issues concerning the greater good in the debate or to discuss soft impacts.

Yet, the participatory process is not only concerned with the inclusion of different groups: it also requires a broadening of the *substantive normative* issues discussed. Although stakeholders and lay publics involved in TA activities are in a position to discuss the desirability of emerging science and technology, the normative dimension of such discussions is often neglected in favour of discussions concerning stakeholders’ *factual acceptance* (Grunwald 1999, p. 175). In the majority of the cases, evaluative exercises on the *normative acceptability* of technologies are dismissed as pertaining to the subjective sphere, one that does not align well with TA’s institutional commitment to provide neutral and objective knowledge. As it has been argued, this is a foundational myth (Torgersen 2019) that should urgently be reconsidered in the face of contemporary politics (Delvenne and Parotte 2019).

### Taking normativity to heart

In this contribution, we distinguish between normativity *of* TA as the overarching moral goal of democratizing decision-making around science and technology (‘meta-normativity’) and normativity *in* TA as the values and moral standards intrinsic in TA (‘in-normativity’). The so-called ‘normative deficit’ in TA, therefore, does not seem to imply that TA lacks a value dimension, as on the contrary such a dimension is visible both at the meta-level and at the level of practice. Instead, it can be understood as a lack of reflexivity within TA practices about their inherent normative inclinations and procedures. Our conceptualization allows us to discuss different types of criticism that emerge in the literature about TA’s lack of acknowledgement for its normative dimensions. Not only the legitimacy of participatory exercises, its effectiveness for democratic purposes and the way consensus around one evaluation is reached require more investigation. TA exercises also tend to reproduce norm-laden distinctions in their set-up (fact/value or expert/lay person) and do not always engage in explicit explorations of participants’ normative stances and moral visions. How can TA initiatives offer robust assessments of new technologies without addressing the very central question of the intrinsic normativity of such evaluations and discussing the moral assumptions that they entail?

*TA institutions would do well to foster this capacity for reflection around procedural and substantive normativities instead of promoting an unrealistic idea of neutrality*.

Amongst others, Grunwald (1999) and Brey (2012) have highlighted that TA activities should be integrated into an applied ethics perspective, which centres on normative aspects. There is also an acknowledgement that rather than adopting a unifying moral theory to evaluate emerging technologies, which is typical of traditional applied ethics approaches, TA initiatives should remain open to a normative pluralism: ethical technology assessment (Palm and Hansson 2006) and the ethical toolbox developed by the Ethical Bio-TA Tools project (Beekman et al. 2006) specifically address the challenge of broadening TA to include moral issues, an exploration of stakeholders’ meanings and visions, the unpacking of their core values and an analysis of their moral arguments.

In order to escape the pitfalls of a normative deficit, TA practitioners would do well to:

1. make visible how actors involved in deliberation actually negotiate the terms of their engagement rather than assuming that deliberation improves the quality of decisions and enhances democracy *per se;*
2. open up discussion among all involved parties on the normativities of deliberative engagement, including the process norms that govern interaction (e. g. reciprocity) and the substantive biases inherent in discourses in and around TA;
3. acknowledge that TA mobilizes both a substantive and procedural normative ideal of good decision-making on science and technology and scrutinize these guiding normative principles.

These recommendations encourage a reflection on a range of normative questions that have methodological, practical, and political implications. TA institutions would do well to foster this capacity for reflection around procedural and substantive normativities instead of promoting an unrealistic idea of neutrality. If such aspects are not taken into account, TA appears to be at odds with its own aims and promises. From the viewpoint of sustaining TA in contemporary knowledge-based economies, TA runs the risk of institutional irrelevance, as when parliamentary TA offices are downsized, or when TA’s proximity to the establishment leads to accusations that TA hampers truly democratic policy-making (van Oudheusden et al 2015; Delvenne and Parotte 2019). What is needed then is the development of a capacity for critical self-reflection on the norms, assumptions, and aims that inform TA agendas, in ways that resonate with TA attempts at bringing reflexivity into science and technology governance.

Even if issues about the good life and ethical perspectives are addressed and taken seriously in TA activities, it remains to be seen how they can be transformed into political action in ways that do justice to the vast plurality of views and concerns. Should policy makers make decisions based on a TA report that restricts itself to spelling out different positions? Alternatively, should TA also propose some positions as better for society from its own normative position? What priority, if any, should normative ethical arguments have over other types of assessment (e. g. economic impact assessment)?

Our article is meant as a contribution to addressing these kinds of questions, and to ongoing debates about the rightful place of science and technology in society, especially in the wake of EU-wide policy agendas. As van Lente and colleagues (2015) point out, these agendas explicitly bring ethics into science and technology-based innovation. We contend that if the collective pursuit of ethical innovation is to prove fruitful, societies should not only critically reflect on the ethics of science and technology, but also on the ethics, visions, and principles that guide, and potentially shape, processes aiming at governing innovation, such as TA.
